# Anti-tumour activity of a first-in-class agent NUC-1031 in patients with advanced cancer: results of a phase I study

**DOI:** 10.1038/s41416-018-0244-1

**Published:** 2018-09-12

**Authors:** Sarah P. Blagden, Ivana Rizzuto, Puvan Suppiah, Daniel O’Shea, Markand Patel, Laura Spiers, Ajithkumar Sukumaran, Nishat Bharwani, Andrea Rockall, Hani Gabra, Mona El-Bahrawy, Harpreet Wasan, Robert Leonard, Nagy Habib, Essam Ghazaly

**Affiliations:** 1 Department of Surgery and Cancer, Hammersmith Campus, Imperial College, London, W12 0HS UK; 2 Early Phase Clinical Trials Unit, Department of Oncology, Churchill Hospital, Headington, Oxford OX3 7LE UK; 3 NIHR/Wellcome Trust Imperial CRF, Imperial Centre for Translational and Experimental Medicine, Hammersmith Hospital, London, W12 0HS UK; 4 Department of Radiology, Imperial College Healthcare NHS Trust, London, W12 0HS UK; 5 Clinical Discovery Unit, Early Clinical Development, AstraZeneca, Cambridge, UK; 6 Imperial College London, London, W12 0HS UK; 7 Centre for Haemato-Oncology, Barts Cancer Institute, Queen Mary University of London, Charterhouse Square, London, EC1M 6BQ UK

**Keywords:** Drug development, Drug development, Drug development, Drug development

## Abstract

**Background:**

Gemcitabine is used to treat a wide range of tumours, but its efficacy is limited by cancer cell resistance mechanisms. NUC-1031, a phosphoramidate modification of gemcitabine, is the first anti-cancer ProTide to enter the clinic and is designed to overcome these key resistance mechanisms.

**Methods:**

Sixty-eight patients with advanced solid tumours who had relapsed after treatment with standard therapy were recruited to a dose escalation study to determine the recommended Phase II dose (RP2D) and assess the safety of NUC-1031. Pharmacokinetics and anti-tumour activity was also assessed.

**Results:**

Sixty-eight patients received treatment, 50% of whom had prior exposure to gemcitabine. NUC-1031 was well tolerated with the most common Grade 3/4 adverse events of neutropaenia, lymphopaenia and fatigue occurring in 13 patients each (19%). In 49 response-evaluable patients, 5 (10%) achieved a partial response and 33 (67%) had stable disease, resulting in a 78% disease control rate. *C*_max_ levels of the active intracellular metabolite, dFdCTP, were 217-times greater than those reported for equimolar doses of gemcitabine, with minimal toxic metabolite accumulation. The RP2D was determined as 825 mg/m^2^ on days 1, 8 and 15 of a 28-day cycle.

**Conclusions:**

NUC-1031 was well tolerated and demonstrated clinically significant anti-tumour activity, even in patients with prior gemcitabine exposure and in cancers not traditionally perceived as gemcitabine-responsive.

## Background

**Fig. 1:**
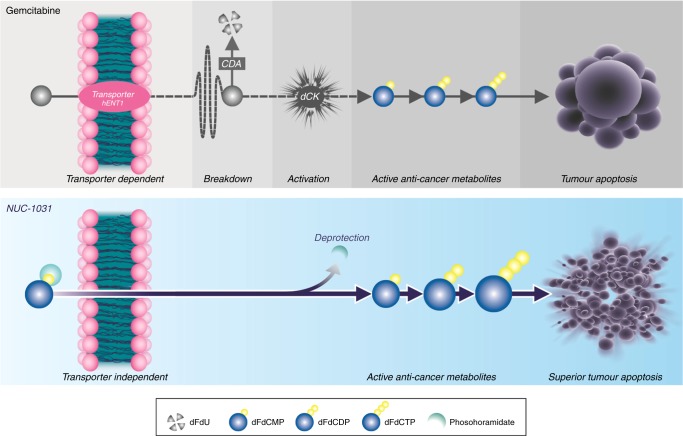
NUC-1031 and gemcitabine mechanism of action. There are three key cancer resistance mechanisms associated with a poor survival prognosis with gemcitabine therapy: transport, activation and breakdown. The transport of gemcitabine, an inactive prodrug, into cancer cells is mediated via hENT1. Once inside the cell, gemcitabine requires phosphorylation to dFdCMP by dCK, which represents the rate-limiting step for further phosphorylation to the active diphosphate (dFdCDP) and triphosphate (dFdCTP) metabolites. Gemcitabine is also rapidly catabolised by CDA generating a metabolite. NUC-1031 was designed to overcome the key cancer resistance mechanisms associated with gemcitabine. NUC-1031 enters the cell independent of the hENT1 transporter and does not require activation by dCK. Similar to the phosphorylated forms of gemcitabine, NUC-1031 is not subject to breakdown by CDA. NUC-1031 is designed to generate and maintain higher concentrations of the anti-cancer metabolite (dFdCTP) inside the tumour compared to gemcitabine

Nucleoside analogues, such as gemcitabine (2′,2′-difluorodeoxycytidine, dFdC), are the backbone of many therapeutic regimens in oncology. However, many cancers have innate or acquired resistance to nucleoside analogues, markedly limiting their efficacy.^1–5^ For gemcitabine, expression of proteins required for transport, activation and/or breakdown of the drug has been correlated with treatment resistance and adverse survival outcome.^6,7^ The transport of gemcitabine into cancer cells is mainly mediated via human equilibrative nucleoside transporter 1 (hENT1). Once inside the cell, gemcitabine requires phosphorylation to difluorodeoxycytidine monophosphate (dFdCMP) by deoxycytidine kinase (dCK), which represents the rate-limiting step for further phosphorylation to the active diphosphate (dFdCDP) and triphosphate (dFdCTP) metabolites. Of these, dFdCTP is the more active and incorporates into DNA to inhibit its synthesis, whilst dFdCDP inactivates ribonucleotide reductase, depleting the deoxyribonucleotide pools necessary for DNA synthesis, potentiating the effects of dFdCTP.^8^ Gemcitabine is also rapidly catabolised by cytidine deaminase (CDA) generating difluorodeoxyuridine (dFdU) (Fig. 1).^2^

Various strategies to counter these treatment-limiting pathways have been investigated, although previous attempts have only considered individual resistance factors rather than the combined effects of all three.^9–11^ To address this clinical challenge, NUC-1031 (Acelarin^®^) was developed as part of a new class of anti-cancer drugs, termed ProTides, whereby the inactive nucleoside analogue prodrug, gemcitabine (dFdC), is converted to dFdCMP and protected by the addition of specific combination of an aryl, ester and amino acid grouping. Pre-clinical data show the increased hydrophobicity of NUC-1031 enables it to circumvent hENT1-mediated transmembrane transport and, once inside the cell, the phosphoramidate protective group is cleaved off by esterases, releasing dFdCMP which is then rapidly converted to dFdCDP and dFdCTP, bypassing the rate-limiting step of dCK phosphorylation. Furthermore, NUC-1031 avoids CDA-mediated catabolism, thus preventing dFdU accumulation (Fig. 1).^12–14^

The ProTide approach has successfully been applied to the development of several approved anti-viral drugs containing nucleoside analogues, including sofosbuvir (Sovaldi^®^) and tenofovir alafenamide fumarate (TAF^®^), which is a key component of Genvoya^®^. However, NUC-1031 (Supplementary Fig. 1) is the first anti-cancer ProTide to enter the clinic. The purpose of this first-in-human study was to assess its safety and anti-tumour activity in patients with advanced solid tumours and define the recommended Phase II dose (RP2D). In addition, pharmacokinetic (PK) analyses were conducted to provide mechanistic proof-of-concept for this first-in-class compound.

## Patients and Methods

### Patient eligibility

Patients ≥18 years of age with a diagnosis of cancer refractory (or not amenable) to standard therapy were eligible for the study. Other inclusion criteria included a life expectancy of at least 12 weeks, Eastern Cooperative Oncology Group (ECOG) performance status^15^ of 0–2 and the following haematological and biochemical parameters: adequate bone marrow (leukocytes ≥ 3 × 10^9^/L, neutrophils ≥ 1.5 × 10^9^/L, platelets ≥ 100 × 10^9^/L, haemoglobin ≥ 9 g/dL), liver function (total bilirubin ≤ 1.5 × upper limit of normal [ULN]), aspartate amino transferase (AST)/alanine amino transferase (ALT ≤ 2.5 × ULN (or ≤ 5 × ULN if liver metastases) and renal function (serum creatinine ≤ 1.5 × ULN).

All patients provided written informed consent. The study was performed in accordance with Good Clinical Practice guidelines and the principles of the 1964 Declaration of Helsinki and subsequent revisions.

### Study design

This was a two-part, open-label, Phase I, dose escalation and expansion study. In Part 1 (dose-escalation), two administration schedules of NUC-1031 were assessed over a 28-day cycle: Schedule A—weekly (days 1, 8 and 15), and Schedule B—twice weekly (days 1, 5, 8, 12, 15 and 19). Six patients were recruited to Schedule B Cohort 3 and received NUC-1031 at 375 mg/m^2^ twice weekly; however, it became apparent that drug administration on Schedule B was logistically challenging for both the patients and the trial site, and further study of Schedule B was discontinued. Thereafter, NUC-1031 was only administered on days 1, 8 and 15 every 28 days. Patients received treatment for six cycles unless there was evidence of clinical or radiological progression, unacceptable toxicity or if they declined further treatment. Patients could receive NUC-1031 beyond Cycle 6 on a compassionate basis. Assessment of toxicity was used to establish the RP2D for the Part 2 expansion cohort.

The primary endpoints were safety and determination of RP2D. Safety was assessed by adverse event profile and changes from baseline in vital signs, clinical laboratory parameters and electrocardiogram (ECG). Secondary endpoints included PK and evaluation of anti-tumour activity. A dose-limiting toxicity (DLT) was defined as one or more events, judged related to NUC-1031, occurring through to the last scheduled day of Cycle 1. These included; Grade ≥3 toxicity (except nausea/vomiting/diarrhoea in the absence of effective therapy), Grade ≥3 nausea/vomiting/diarrhoea that occurs despite standard medical treatment, Grade 4 neutropaenia or thrombocytopaenia, febrile neutropaenia, or inability to begin next dose of treatment within 14 days of scheduled dosing due to unresolved toxicity relating to NUC-1031. The study protocol was approved by the Medicines and Healthcare products Regulatory Agency, the West London Research Ethics Committee 12/LO/1100 and local review boards. The study was registered on clinicaltrials.gov (NCT01621854).

### Patient evaluation

All patients receiving at least one dose of NUC-1031 were evaluable for toxicity. Only patients who had measurable disease and had received at least two cycles of treatment were considered response-evaluable. Patients who did not complete the first cycle of treatment for reasons other than DLTs were not evaluable for the maximum tolerated dose (MTD) and were replaced. Adverse event data were collected from the start of study drug administration until 30 days after the last dose was administered and graded using National Cancer Institute (NCI) Common Terminology Criteria for Adverse Events (CTCAE) Version 4.02. Laboratory safety assessments and vital signs were performed on dosing days from baseline to end of study visit.

Tumours were assessed by computed tomography (CT) or magnetic resonance imaging (MRI) scan after Cycles 2, 4 and 6 and compared with those conducted at study entry using Response Evaluation Criteria in Solid Tumours (RECIST) 1.1 criteria. Efficacy was determined in all patients with measurable disease at baseline and who completed at least two cycles of NUC-1031 and had at least one follow-up radiographic assessment to measure changes in tumour size. The best overall response was defined as best response on at least one time point; results were displayed in a waterfall plot. Progression-free survival (PFS) was measured from the first administration of NUC-1031 to disease progression or death (whichever occurred first). Duration of response was calculated from date of first response until RECIST or symptomatic disease progression.

### Dose modifications and delays

Treatment interruptions of up to 14 days were permitted in order for participants to meet the re-treatment criteria before starting their next cycle.

### Pharmacokinetic analysis

Blood samples were collected for PK analysis during Cycle 1 on days 1 and 15 pre-dose and at numerous time points after the end of infusion (0.217, 0.3, 0.467, 0.633, 1, 1.5, 2, 4, 6 and 24 h). Blood (6 mL) was collected using heparinised blood collection tubes spiked with tetrahydrouridine (25 μg/mL) to inhibit CDA activity. Plasma was separated by centrifugation and peripheral blood mononuclear cells (PBMCs) were separated using Lymphoprep density gradient (STEMCELL Technologies UK Ltd). PBMCs were extracted and assayed for dFdCMP, dFdCDP and dFdCTP using a previously described liquid chromatography-tandem mass spectrometry (LC-MS/MS) method.^16^ Plasma and urine samples were assayed for NUC-1031, dFdC and dFdU. (See Supplementary Methods for detailed protocol.)

### Statistical analysis

Sample size calculations were based on a Fleming design.^17^ No formal statistical analyses were planned or performed on safety, PK or efficacy data.

## Results

### Patient characteristics

**Fig. 2:**
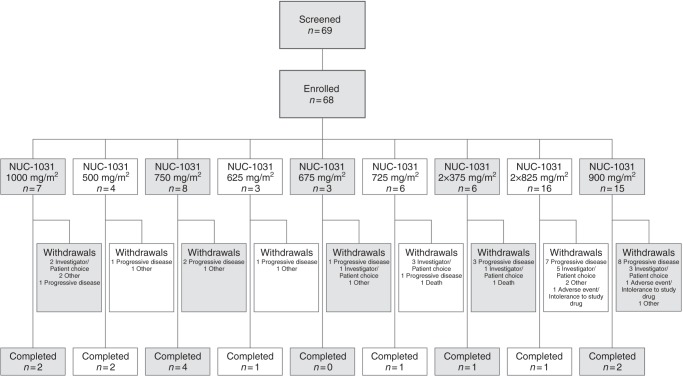
CONSORT flow chart. Sixty-eight patients with advanced solid malignancies were enrolled into the study and were safety-evaluable. In Schedule A, patients received doses of 500 mg/m^2^, 625 mg/m^2^, 675 mg/m^2^, 725 mg/m^2^, 750 mg/m^2^, 825 mg/m^2^, 900 mg/m^2^ and 1000 mg/m^2^ (all given once-weekly). In Schedule B, patients received a dose of 375 mg/m^2^ twice weekly. The most common reasons for withdrawal were progressive disease (25 patients) and Investigator/patient choice (15 patients). Overall, 14 patients completed the study (received ≥ 6 cycles)

A total of 68 patients with advanced solid malignancies were enrolled into the study between 5 October 2012 and 24 June 2015 (Fig. 2) and were evaluable for safety analysis. Forty-six were female and 22 were male, patients had a mean age of 56 years (range 20–83 years; Table 1). Overall, there were 19 primary cancer types, the most frequent being ovarian (*n* = 10, 15%), pancreas (*n* = 9, 13%), biliary (*n* = 7, 10%) and colorectal (*n* = 7, 10%). Patients had an average of three previous chemotherapy regimens (range 1–8); 34 (50%) had received prior gemcitabine chemotherapy.

**Table 1:** Demographic and clinical characteristics at baseline

Characteristic	Study population (*n* = 68)
Sex; *n*	
Female	46
Male	22
Age, years; mean [range]	56.3 [20–83]
Ethnicity; *n* (%)	
White	49 (72.1)
Black or black British	8 (11.8)
Asian or Asian British	7 (10.3)
Other	3 (4.4)
Mixed	1 (1.5)
Body mass index, kg/m^2^; mean (standard deviation)	25.4 (5.13)
ECOG performance score; *n* (%)	
0	26 (38.2)
1	38 (55.9)
2	4 (5.9)
Previous chemotherapy regimens^a^; mean [range] *n*	3.0 [1–10] 68
Previous treatment with gemcitabine; *n* (%)	34 (50)
Primary cancer; *n* (%)	
Ovarian/fallopian tube	13 (19)
Pancreatic	9 (13)
Cholangiocarcinoma	7 (10)
Colorectal	7 (10)
Non-small cell lung	6 (9)
Breast	4 (6)
Endometrial	3 (4)
Mesothelioma	3 (4)
Oesophageal	3 (4)
Unknown primary	3 (4)
Cervical	2 (3)
Gastric	1 (2)
Kidney	1 (2)
Osteosarcoma	1 (2)
Small cell lung	1 (2)
Anal	1 (2)
Thymus	1 (2)
Adrenal	1 (2)
Mixed trophoblastic tumour (PSTT/ETT)	1 (2)
Stage at initial diagnosis; *n* (%)	
Stage I	2 (3)
Stage II	7 (10)
Stage III	8 (12)
Stage IV	29 (43)
Unknown	22 (32)

*ECOG* Eastern Cooperative Oncology Group, *PSTT* placental site trophoblastic tumour, *ETT* epithelioid trophoblastic tumour

^a^Includes cytotoxic treatments only; does not include radiotherapy, hormone therapies or targeted therapies

### Dose determination and MTD

The dose levels studied, the number of patients treated and AEs are summarised in Table 2. During schedule A, the following doses of NUC-1031 were administered: 500 mg/m^2^, 625 mg/m^2^, 675 mg/m^2^, 725 mg/m^2^, 750 mg/m^2^, 825 mg/m^2^, 900 mg/m^2^ and 1000 mg/m^2^ (all given once-weekly, for 3 weeks out of every 4-week cycle). The MTD for schedule A was defined as 1000 mg/m^2^ following DLTs of Grade 4 neutropaenia, thrombocytopaenia and posterior reversible encephalopathy syndrome (PRES) in one patient and a second patient with two separate DLTs of Grade 3 hepatic transaminitis. A DLT of Grade 4 thrombocytopaenia was seen in one patient receiving 750 mg/m^2^, and two DLTs of transient Grade 3 hepatic transaminitis were seen in a patient in the 725 mg/m^2^ cohort. Schedule B (375 mg/m^2^ twice weekly) was administered to six patients, but the visit frequency was considered logistically challenging for patients and no further doses were explored within this schedule. The dose of 825 mg/m^2^ (schedule A) was selected for the expansion (Part 2) of the study, and 12 patients were recruited at this dose, in addition to the four who received this dose in Part 1.

**Table 2:** Summary of adverse event grades and types

	NUC-1031 dose (mg/m^2^)								
*n* in dose cohort (*N* = 68)	500 (4)	625 (3)	675 (3)	725 (6)	2 × 375^a^ (6)	750 (8)	825 (16)	900 (15)	1000 (7)
	*n* (%)	*n* (%)	*n* (%)	*n* (%)	*n* (%)	*n* (%)	*n* (%)	*n* (%)	n (%)
SAEs	2 (50)	2 (66.7)	1 (33.3)	6 (100)	4 (67)	6 (75)	8 (50)	10 (66.6)	5 (71.4)
AEs (all Grades)	4 (100)	3 (100)	3 (100)	6 (100)	6 (100)	8 (100)	16 (100)	15 (100)	7 (100)
AEs (Grade 3/4)	3 (75)	2 (66.7)	2 (66.7)	6 (100)	6 (100)	8 (100)	14 (87.5)	12 (80.0)	7 (100)
Withdrawn due to AE	1 (25)	1 (33.3)	0	1 (16.7)	1 (16.7)	1 (12.5)	6 (37.5)	3 (20.0)	1 (14.3)
Chemistry AE^b^ Grade 3	1 (25)	2 (66.7)	1 (33.3)	3 (50)	3 (50)	5 (62.5)	7 (43.8)	8 (53.3)	4 (57.1)
Chemistry AE^b^ Grade 4	0	1 (33.3)	0	0	0	0	0	0	0
Haematological AE^c^ Grade 3	1 (25)	1 (33.3)	0	4 (66.7)	4 (66.7)	6 (75)	5 (31.3)	8 (53.3)	3 (42.9)
Haematological AE^c^ Grade 4	0	0	0	0	2 (33.3)	2 (25)	1 (6.3)	0	1 (14.3)

^a^375 mg/m^2^ was administrated twice-weekly in 6 patients (Schedule B)

^b^Chemistry AEs included: ALT increased, hypoalbuminaemia, blood bilirubin increased, hypokalaemia, hyponatraemia, hypophosphataemia, blood urea increased, hypomagnesaemia, blood creatinine increased, hyperglycaemia, blood albumin decreased, AST increased, blood alkaline phosphatase increased, blood glucose increased, blood phosphorous decreased hypocalcaemia, hyperkalaemia

^c^Haematological AEs included: neutropaenia, thrombocytopaenia, white blood cell count decreased, platelet count decreased, neutrophil count decreased, anaemia, lymphocyte count decreased, leukopaenia

The dose intensity (DI; the proportion of the scheduled NUC-1031 dose that was successfully administered to the patient within the first 8 weeks of study) was measured for each cohort and the results are shown in Supplementary Table 1. In dose cohorts up to and including 825 mg/m^2^ it was possible, on average, to administer >70% of the calculated total dose but the percentage fell as the dose increased above 825 mg/m^2^. At 825 mg/m^2^, 58% of patients achieved 100% DI and 75% of patients achieved ≥75% DI. When the actual DI was at or close to the targeted DI there was a high proportion of disease control as shown in Supplementary Table 2.

### Safety and tolerability

NUC-1031 was administered as a short (10–15 min) intravenous infusion via central venous access devices. Forty-four of the 68 patients experienced 94 treatment-emergent serious adverse events (SAEs; Table 2), 27 of which were Grade 3/4 and judged to have a definite, probable or possible relationship to NUC-1031. Six (22%) of the Grade 3/4 related SAEs to be reported by more than one patient were increased alanine amino transferase (*n* = 4), pyrexia (*n* = 3), thrombocytopaenia (*n* = 3), hypoxia (*n* = 2), lung infection (*n* = 2) and neutropaenia (*n* = 2). Fatigue and transaminitis (56 patients each, 82.4%) were the most frequently reported adverse events, irrespective of grade or relationship. Neutropaenia, lymphopaenia and fatigue were the most frequently reported Grade 3/4 adverse events (13 patients each, 19.1%) (Supplementary Table 3).

Six patients died during the study, events that were judged to be unrelated or unlikely to be related to NUC-1031. Of note, 9 (13.3%) patients had pulmonary embolism, 5 of which were incidental radiological findings. Of the sixty-eight patients, 17 (25%) received a complete six-cycle course of study treatment. Fourteen patients opted to continue on NUC-1031 beyond Cycle 6, and received up to 13 additional treatment cycles (Fig. 2).

### Pharmacokinetics

Sixty-six patients had evaluable PK samples. As this was a Phase I study with ascending dose schedules and variability in the plasma concentration-time profiles there was some imprecision in AUC_0-inf_ and *t*_1/2_ estimates. The most robust comparisons can be made using AUC_0-*t*_ and *C*_max_. Nevertheless, after administration, NUC-1031 was detected in the plasma up to 24 h from End Of Infusion (EOI) with an estimated *t*_1/2_ of 8.3 h, which is considerably longer than the reported *t*_1/2_ of gemcitabine (2.3–80 min).^18–21^

Among the analytes evaluated, median plasma AUC_0-*t*_ and *C*_max_ estimates on day 1 were highest for NUC-1031 (269 µM/h and 710 µM, respectively), intermediate for dFdU (76.0 µM/h and 5.11 µM, respectively) and lowest for dFdC (2.92 µM/h and 1.82 µM, respectively).

Of note, there were no trends for increased exposures on Cycle 1, day 15 indicating that systematic accumulation of NUC-1031, dFdC or dFdU concentrations does not occur with either once- or twice-weekly dosing of NUC-1031. Plasma PK data are summarised in Supplementary Table 4. Dose proportionality was only observed for NUC-1031 on Cycle 1, day 1. The lack of dose proportionality is not unexpected given that the study was not designed to show dose proportionality and most of the dose levels contained less than 10 patients.

**Fig. 3:**
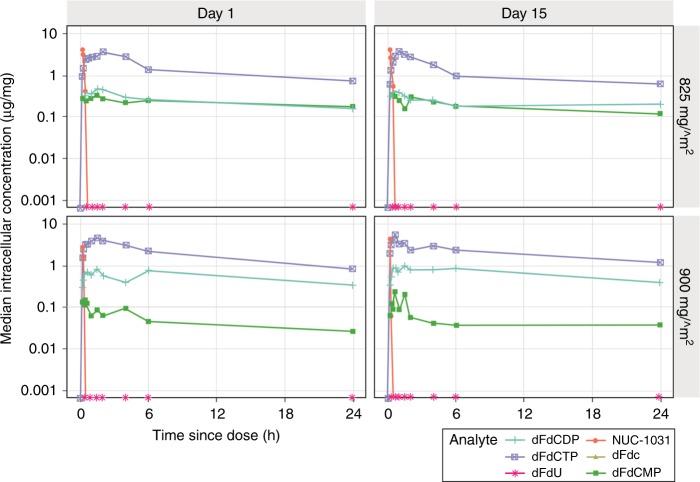
Median intracellular analyte concentrations. Median intracellular analyte concentrations at scheduled sampling days were stratified by dose and day and plotted on a semi-log scale (825 and 900 mg/m^2^ only). Intracellular concentrations were all normalised to tissue protein concentrations. Intracellular concentrations of the active anti-cancer moiety dFdCTP remained high throughout the 24-h PK sampling window

Intracellular concentrations of the active anti-cancer moiety dFdCTP remained high throughout the 24-h PK sampling window (Fig. 3). Mean intracellular dFdCTP AUC_0-*t*_ and *C*_max_ estimates in patients PBMCs on day 1 normalised to a dose of 500 mg/m^2^ were 151,413 µM/h and 26,910 µM, respectively (Supplementary Table 5).

Analysis of urine samples from 46 patients demonstrated that 21.7 and 27.3% of the NUC-1031 was excreted via the urine as dFdU over the 24 h after the dose on days 1 and 15, respectively. Overall, less than 1% of the dose was excreted as either NUC-1031 or dFdC, suggesting that the ProTide is stable during plasma transport to the tumour cells.

### Efficacy

Of the 68 patients participating in the study, 19 (8 males:11 females; average age 56 years; range 35–73) were not evaluable for efficacy of NUC-1031; two patients died of causes unrelated to study drug, four patients developed progressive disease before completing two cycles, and the remainder withdrew from the study for a variety of reasons (see Fig. 2). The average number of treatment cycles with NUC-1031 in these non-evaluable patients was 0.8 cycles (range 0.3–1.6 cycles).

**Fig. 4:**
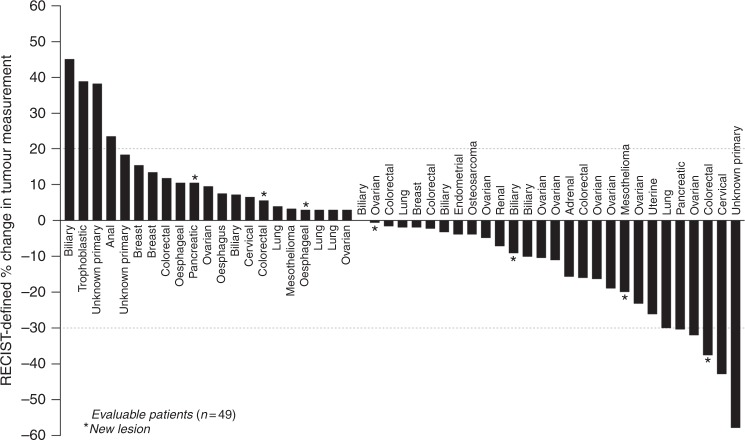
Waterfall plot of best response to therapy. Forty-nine patients received ≥ 2 cycles of NUC-1031 and had a scan for assessment of efficacy. Clinical activity was achieved across 19 primary cancer types, the most frequent being ovarian, pancreatic, biliary and colorectal. Eleven patients had progressive disease and the best overall responses were five PRs (10%) and 33 SDs (67%). Of the 33 SDs, 12 (24%) were of at least 6 months duration

The 49 patients who received ≥2 cycles of NUC-1031 and had a scan for assessment of efficacy received an average of 4.8 cycles (range 2–19 cycles) with a median PFS of 4.0 months (range 1–25 months). Eleven patients had progressive disease and the best overall responses in the evaluable patients were 5 partial responses (PRs) (10%) and 33 stable diseases (SDs) (67%; Table 3). Of the 33 SDs, 12 (24%) were of at least 6 months duration. Five (10%) patients with primary cancers of the cervix, lung, fallopian tube, pancreas and unknown primary achieved PRs (Table 3; Fig. 4). Two of these five patients had subsequent scans confirming their partial response. Of 16 patients who had previously progressed on or following a gemcitabine-containing regimen, one patient achieved a PR and nine patients had SD.

**Table 3:** Anti-tumour activity of NUC-1031

*n* in dose cohort	NUC-1031 dose mg/m^2^								
	500	625	675	725	2 × 375^a^	750	825	900	1000
	(2)	(2)	(1)	(5)	(5)	(7)	(12)	(11)	(4)
Complete response - *n*	0	0	0	0	0	0	0	0	0
Partial response (PR) - *n* (95% CI)	0	1 (9.5,90.5)	0	0	0	3 (15.8,75.0)	0	0	1 (4.6,69.9)
Confirmed PR - *n* (%)	NA	0	NA	NA	NA	2 (28.6)	NA	NA	NA
Stable disease - *n*	2	1	0	5	5	2	9	6	3
(95% CI)	(34.2,100.0)	(9.5,90.5)		(56.6,100.0)	(37.6,96.4)	(8.2,64.1)	(46.8,91.1)	(28.0,78.)	(30.1,95.4)
Progressive disease - *n*	0	0	1	0	0	2	3	5	0
Disease control rate - *n* (%)	2 (100)	2 (100)	0	5 (100)	5 (100)	5 (71.4)	9 (75)	6 (54.5)	4 (100)
Progression-free survival (months)									
Censored, *n*	2	2	0	2	2	3	5	2	2
Events, *n*	0	0	1	3	3	4	7	9	2
Mean (SD)	9.2 (3.09)	5.2 (1.12)	1.8	4.0 (2.32)	3.2 (2.73)	7.7 (8.36)	3.6 (1.83)	3.6 (1.77)	5.5 (2.72)
Median	9.2	5.2	1.8	3.5	3.1	5.3	3.3	3.7	5.2
Range	7.0–11.3	4.4–6.0	NA	1.6–7.9	0.5–7.5	1.5–25.0	1.6–8.3	1.5–7.1	2.8–8.8

CI, confidence interval; SD, standard deviation.

^a^375 mg/m^2^ was administrated twice-weekly in 6 patients (Schedule B)

## Discussion

We report results from the first-in-human study of NUC-1031 belonging to a new class of anti-cancer agents called ProTides that are designed to improve the efficacy and safety profile of conventional nucleoside analogues. NUC-1031, a chemical modification of gemcitabine, is the first anti-cancer ProTide to enter the clinic. In this Phase I setting of heavily pre-treated patients with advanced solid tumours, NUC-1031 achieved good disease control with an acceptable safety profile. There were no unexpected adverse events and the most common adverse reactions were similar to those observed with gemcitabine^22^ and included reversible myelosuppression, gastrointestinal disturbances, fatigue and elevations in liver function enzymes. At or below the RP2D of 825 mg/m^2^, NUC-1031 could be administered at high dose intensity, which corresponded with a more favourable clinical outcome. Ten percent of evaluable patients achieved responses of PR, and SD was observed in a further 67%, resulting in an overall Disease Control Rate of 78%. Responses were durable, the median PFS was 4 months (censored). Notably, responses were observed amongst patients whose tumours had progressed during or following prior gemcitabine therapy. This is in line with pre-clinical data that demonstrated NUC-1031 has activity in the context of gemcitabine resistance.

NUC-1031 has a more favourable PK profile than gemcitabine. PK data confirmed higher intracellular concentrations of the active anti-cancer metabolite dFdCTP (Supplementary Table 5), with 217- and 139-fold-higher respective *C*_max_ and AUC_0-*t*_, than have been reported with gemcitabine at equimolar doses.^18–21,23,24^ Whilst these results cannot be directly translated from PBMCs to tumour cells they do suggest that, following NUC-1031 administration, tumour cells are exposed to considerably higher levels of the active metabolite dFdCTP than have been observed following gemcitabine. Furthermore, it was noted that NUC-1031 administration resulted in prolonged high intracellular concentrations of dFdCTP throughout the subsequent 24-h period than are observed at the *C*_max_ 2-h time point after gemcitabine administration. The estimated NUC-1031 plasma *t*_1/2_ of 8.3 h compared favourably with the (shorter) reported gemcitabine plasma *t*_1/2_ of 2.3–80 min and confirms the insensitivity of NUC-1031 to CDA-mediated plasma deamination.^18–21^ The longer half-life of NUC-1031 therefore could enable tumour cells to receive a more prolonged exposure to dFdCTP which may enhance its activity, paralleling reports that the anti-tumour activity of gemcitabine is improved using longer infusion times (up to 24 h).^25^ Furthermore, following exposure to NUC-1031, there were considerably lower plasma and intracellular levels of the metabolite dFdU than have been reported with gemcitabine, which may explain the acceptable toxicity profile observed with NUC-1031.

Maintaining the dose intensity is an important factor in achieving optimum benefit for any anti-cancer agent.^26,27^ Patients who received NUC-1031 at a dose at or below 825 mg/m^2^ were generally able to maintain treatment intensity (i.e. receive subsequent infusions when planned); doses of 900 mg/m^2^ and above required more substantial dose optimisation and scheduling due to cumulative fatigue and myelosuppression. Notably, four of the five partial responses occurred in patients who received a doses at or below 825 mg/m^2^. A limitation of the study was the inability to fully assess PFS because the initial study design did not include any post-treatment follow-up and was censored at 6 months. Thus, the PFS data reported here might underestimate survival.

In conclusion, NUC-1031 is the first of a group of protected nucleotide analogues designed to counter the key cancer resistance mechanisms associated with gemcitabine therapy. NUC-1031 achieved clinical activity across multiple tumour types, even in cancers that are not traditionally perceived as gemcitabine-responsive. This, along with observed activity in patients who were previously exposed to gemcitabine, indicates a broad spectrum of activity for NUC-1031 which is likely to be attributable to its ability to bypass cellular resistance mechanisms. NUC-1031 was well tolerated with no unexpected adverse events and an acceptable safety profile that allowed prolonged administration for many months (up to 20 months in one patient). Although the dose of 825 mg/m^2^ once-weekly for 3 weeks in a 4-weekly cycle was selected for further evaluation as monotherapy, it was noted that doses of 500 mg/m^2^ and higher also showed evidence of clinical activity and warrant further study. Clinical studies of NUC-1031, either as monotherapy or in combination with other agents, are currently ongoing in patients with ovarian, biliary and pancreatic cancers.

## Electronic supplementary material

Supplementary Methods, Tables and Figures

## Availability of data and materials

The datasets used and analysed during the current study are available from the corresponding author on reasonable request.
