# Supplementary material for: Anti-tumour activity of a first-in-class agent NUC-1031 in patients with advanced cancer: results of a phase I study
Source: Br J Cancer. 2018 Sep 12;119(7):815–22. doi: 10.1038/s41416-018-0244-1 (PMC6189138; doi:10.1038/s41416-018-0244-1)
Supplement: Supplementary file 1 — Supplementary Methods, Tables and Figures [file 41416_2018_244_MOESM1_ESM.docx]

**Supplementary Methods, Tables and Figures**

| **Supplementary Methods** | Detailed pharmacokinetic (PK) methodology |
| --- | --- |
| **Supplementary Table 1** | Dose intensity achieved within the first 8 weeks of NUC-1031 administration |
| **Supplementary Table 2** | Proportion of evaluable patients receiving 100% of calculated dose and related to effect on disease status |
| **Supplementary Table 3** | Summary of grade 3 or grade 4 adverse events |
| **Supplementary Table 4** | Summary of plasma PK concentrations |
| **Supplementary Table 5** | Comparison of observed dFdCTP exposure in PBMCs in patients enrolled in PRO-001 to published data |
| **Supplementary Figure 1** | Chemical structures of NUC-1031 and gemcitabine |

**Supplementary Methods.** Detailed pharmacokinetic (PK) methodology

Plasma samples were extracted using protein precipitation achieved by addition of methanol (75:25, v/v) containing the internal standards (deuterated NUC-1031 [D5-NUC-1031] for NUC-1031 and deoxycytidine [DC] for dFdC and dFdU). Then, samples were centrifuged and the supernatant was evaporated to dryness and reconstituted in 10% ACN. Urine samples were diluted using 20% ACN (50:50, v/v).

The reconstituted plasma extracts and diluted urine samples were injected on the UPLC‑MS/MS system (TSQ Vantage coupled to Accela UPLC, Thermo Scientific, UK). Analytes were resolved using ACE Ultracore 2.5 µm, Super C18, 100 × 2.1 mm column and a mobile phase consisting of a mixture of water containing 10mM NH_4_Ac (A), and acetonitrile (B). Detection was performed in positive and negative ion modes using the following transitions, NUC-1031: +ve m/z 581.2 → 246.2, D5-NUC-1031: +ve m/z 586.2 → 246.2, dFdU: -ve m/z 263.1 → 221.2, dFdC: +ve m/z 264.1 → 112.2 and DC: +ve m/z 228.1 → 112.2. The retention times for DC, dFdC, dFdU, NUC-1031 and D5-NUC-1031 were 1.8, 2.2, 3.5, 4.8 and 4.8 minutes, respectively. The limit of quantification (LOQ) was 30 ng/mL for all analytes. Linearity of the calibration curve was determined by plotting peak area ratio of the three analytes to internal standard against the analyte concentrations using weighted (1/x^2^) linear regression analysis. A linear response was obtained for the three analytes with R^2^ >0.999. Within-day imprecision was ≤8.2% and between-day imprecision ≤5.3%. Inaccuracy was ≤10.4% of the nominal values of two spiked quality control samples.

The PK parameters were determined using noncompartmental analysis using the software program Kinetica version 3.0 (Thermo Scientific, Philadelphia, PA). Concentration-time data were imported using Microsoft Excel 2013 software (version 15.0.4569.1504) into Kinetica for the analysis. The following PK parameters were calculated: maximum observed concentration (C_max_), time of maximum observed concentration (T_max_), area under the concentration-time curve from time zero to time of last measurable concentration, using log trapezoidal rule (AUC_0-t_) and elimination half-life (t_½_).

To calculate the intracellular C_max_ of dFdCTP in PBMCs following NUC-1031 treatment, μg/mg protein was converted to µmoles per mg of tissue protein using the molecular weight of 503.137, and then converted to µM using the density of protein (1.22 g/cm^3^ [28]), and multiplied by 1000 mL per L. To calculate the intracellular AUC of dFdCTP in PBMCs following NUC-1031 treatment, μg/mg protein was converted to µmol/hr per mg of tissue protein using the molecular weight of 503.137, and then converted to µM/hr using the density of tissue protein (1.22 g/cm^3^ [28]), and multiplied by 1000 mL per L.

As an exploratory endpoint, the plasma PK parameters of AUC_0-t_, AUC_0-inf_, and C_max_ were compared across each dose level to assess dose proportionality (i.e., proportionality of a change in systemic exposure with a change in dose) for each evaluable analyte. Statistical analyses were conducted using a power model with mixed effects of the following general form:

ln(PK) = ln(β0) + β1 / ln(Dose) + ε

where,

- PK is the pharmacokinetic parameter tested (e.g., C_max_ or AUC),
- ln(β0) is the y-intercept,
- β1 is the slope (a value of β1 ~ 1 indicates linearity), and
- ε is an error term (patient was used as the random effects term).

**Supplementary Table 1.** Dose intensity achieved within the first 8 weeks of NUC-1031 administration for evaluable patients in each dose cohort

| Dose range | *n* patients | Average % DI | 100% DI | ≥75% DI |
| --- | --- | --- | --- | --- |
| (mg/m^2^) | (62) | (Range) |  |  |
| 500 | 4 | 77 (50–100) | 50 | 50 |
| 625–675 | 6 | 72 (33–100) | 50 | 50 |
| 725–750 | 14 | 88 (17–100) | 58 | 93 |
| 825 | 16 | 84 (33–100) | 58 | 75 |
| 900 | 15 | 69 17–100) | 20 | 54 |
| 1000 | 7 | 46 (17–95) | 0 | 29 |

DI, dose intensity

**Supplementary Table 2.** Proportion of evaluable patients receiving 100% of calculated dose and related to effect on disease status (expressed as a percentage of total number of patients who received dose)

| Dose range | *n* patients | Patients with 100% DI | Patients with PR/SD |
| --- | --- | --- | --- |
| (mg/m^2^) | (62) | *(n)* | *n* (% of total patients who received dose) |
| 500 | 4 | 2 | 2 (50) |
| 625–675 | 6 | 3 | 2 (34) |
| 725–750 | 14 | 8 | 6 (43) |
| 825 | 16 | 7 | 6 (38) |
| 900 | 15 | 3 | 1 (7) |
| 1000 | 7 | 0 | 0 (0) |

DI, dose intensity; PR, partial response; SD, stable disease

**Supplementary Table 3.** Summary of grade 3 or grade 4 adverse events reported in more than one patient

| **Adverse event** | **Safety population (*n*=68)**  ***n* (%)** |
| --- | --- |
| **General**:  Fatigue | 13 (19.1) |
| Pyrexia | 2 (2.9) |
| **Haematologic:**  Lymphopaenia | 13 (19.1) |
| Leukopaenia | 11 (16.2) |
| Neutropaenia | 13 (19.1) |
| Anaemia | 8 (11.8) |
| Thrombocytopaenia | 9 (13.2) |
| **Metabolic:**  Hypokalaemia | 5 (7.4) |
| Hypophosphataemia | 8 (11.8) |
| Hyponatraemia | 12 (17.6) |
| Hypomagnesaemia | 4 (5.9) |
| Hypoalbuminaemia | 13 (19.1) |
| Hyperglycaemia | 4 (5.9) |
| **Gastrointestinal:**  Abdominal pain | 4 (5.9) |
| Elevated alanine aminotransferase (ALT) | 8 (11.8) |
| Elevated aspartate aminotransferase (AST) | 3 (4.4) |
| Elevated alkaline phosphatase (ALP) | 2 (2.9) |
| Elevated bilirubin | 2 (2.9) |
| Ascites | 3 (4.4) |
| Anorexia | 3 (4.4) |
| Diarrhoea | 3 (4.4) |
| Vomiting | 3 (4.4) |
| Nausea | 2 (2.9) |
| Small intestinal obstruction | 2 (2.9) |
| **Respiratory:**  Respiratory infection | 9 (13.2) |
| Hypoxia | 2 (2.9) |
| **Musculoskeletal:**  Back pain | 4 (5.9) |
| Arthralgia | 3 (4.4) |
| Bone pain | 2 (2.9) |
| **Cardiovascular:** Pulmonary embolism  Hypotension | 9 (13.3)  2 (2.9) |
| Device-related infection | 3 (4.4) |
| **Neurologic:**  Depressed level of consciousness | 2 (2.9) |

**Supplementary Table 4. Summary of plasma PK concentrations**

| Time  (h) | **Plasma concentration (µM)** | | | | | |
| --- | --- | --- | --- | --- | --- | --- |
|  | NUC-1031 (SE)  [NOALQ] | | dFdC (SE)  [NOALQ] | | dFdU (SE)  [NOALQ] | |
|  | Day 1 | Day 15 | Day 1 | Day 15 | Day 1 | Day 15 |
| 0 | 0.00 (0.00)  [0] | 0.00 (0.00)  [0] | 0.00 (0.00)  [0] | 0.00 (0.00)  [0] | 0.00 (0.00)  [0] | 0.00 (0.00)  [0] |
| 0.217 | 710.44 (86.70) [58] | 388.76 (74.04) [51] | 1.82  (0.46)  [57] | 1.79  (0.46)  [52] | 0.49  (0.15)  [55] | 1.10  (0.42)  [50] |
| 0.3 | 353.79 (40.64) [60] | 238.18 (47.50) [52] | 1.22  (0.15)  [59] | 1.71  (0.34)  [53] | 0.68  (0.19)  [58] | 1.63  (0.49)  [53] |
| 0.467 | 190.29 (25.86) [61] | 98.87 (13.47) [50] | 1.25  (0.15)  [60] | 1.18  (0.11)  [52] | 1.29  (0.42)  [60] | 1.85  (0.49)  [52] |
| 0.633 | 115.89 (17.05) [60] | 63.72 (9.94)  [49] | 1.18  (0.11)  [61] | 1.06  (0.11)  [53] | 1.93  (0.45)  [61] | 2.31  (0.57)  [53] |
| 1 | 52.51 (11.66) [63] | 20.97 (3.36)  [49] | 0.61  (0.08)  [65] | 0.65  (0.08)  [53] | 2.76  (0.53)  [65] | 3.07  (0.64)  [53] |
| 1.5 | 28.03 (8.37)  [58] | 10.49 (1.45)  [49] | 0.34  (0.04)  [61] | 0.42  (0.04)  [53] | 3.37  (0.57)  [61] | 3.56  (0.64)  [53] |
| 2 | 17.66 (4.03)  [61] | 6.32  (0.91)  [49] | 0.27  (0.04)  [65] | 0.27  (0.04)  [53] | 3.52  (0.57)  [65] | 4.16  (0.79)  [52] |
| 4 | 6.36  (1.71)  [60] | 2.26  (0.64)  [45] | 0.19  (0.04)  [62] | 0.19  (0.04)  [49] | 4.24  (0.72)  [64] | 4.05  (0.68)  [52] |
| 6 | 4.26  (1.71)  [55] | 1.24  (0.33)  [44] | 0.19  (0.04)  [53] | 0.15  (0.04)  [44] | 5.03  (0.95)  [60] | 4.32  (0.64)  [50] |
| 24 | 1.40  (0.40)  [47] | 0.34  (0.09)  [33] | 0.08  (0.04)  [47] | 0.08  (0.00)  [43] | 5.11  (1.14)  [60] | 3.86  (0.64)  [50] |

NOALQ, number of observations above lower limit of quantification; h, hours; SE, standard error

**Supplementary Table 5.** Comparison of observed dFdCTP exposure in PBMCs in patients enrolled in PRO-001 to published data

| **Reference** | **Gemcitabine dose (mg/m^2^)** | **Inf. time (h)** | **N** | **Median C_max_ (µM)** | **C_max_ at 500 mg/m^2^ (µM)^a^** | **Median AUC (µM/hr)** | **AUC at 500 mg/m^2^ (µM/hr)^a^** |
| --- | --- | --- | --- | --- | --- | --- | --- |
| Grunewald 1990 | 800 | 1 | 4 | - | - | 140 | 87.5 |
|  | 800 | 2 | 4 | - | - | 217 | 136 |
|  | 800 | 3 | 2 | - | - | 432 | 270 |
| Abbruzzese 1991^b^ | 350 | 0.5 | 3 | 284 | 406 | - | - |
|  | 525 |  | 2 | 217 | 207 | - | - |
|  | 790 |  | 4 | 87 | 55 | - | - |
|  | 1000 |  | 5 | 224 | 112 | - | - |
| Grunewald 1992 | 1200 | 2 | 5/4 | 385 | 160 | 2671 | 981 |
|  | 1500 | 2.5 | 5 | 374 | 125 | 3200 | 1112 |
|  | 2400 | 4 | 2/1 | 604 | 126 | 15339 | 3196 |
|  | 3600 | 6 | 2 | 761 | 106 | 8707 | 1209 |
|  | 4800 | 8 | 3 | 600 | 62.5 | 10998 | 1146 |
|  | 6400 | 11.7 | 3 | 862 | 67.3 | - | - |
| Cattel 2006 | 300 | 1 | 1 | 35.8 | 59.7 | 227 | 378 |
|  | 300 | 2 | 1 | 50.1 | 83.5 | 408 | 680 |
|  | 300 | 3 | 1 | 68.6 | 114 | 536 | 893 |
|  | 1000 | 0.5 | 1 | 26.4 | 13.2 | 285 | 143 |
| Peters 2007 | 960 | 0.5 | 1 | 316^c^ | 164 | 284 | - |
| **Mean of literature values** | | | | | **124** | **-** | **1092** |
|  | **NUC-1031 dose (mg/m^2^)** | **Inf. time (h)** | **N** | **Median C_max_ (µM)^d^** | **C_max_ at 500 mg/m^2^ (µM)^e^** | **Median AUC (µM/hr)^f^** | **AUC at 500 mg/m^2^ (µM/hr)^e^** |
| PRO-001 | 375 | 0.17 – 0.5 | 6 | 22372 | 65786 | 66001 | 194080 |
|  | 500 |  | 4 | 8023 | 17694 | 65710 | 144918 |
|  | 625 |  | 3 | 9913 | 17491 | 43895 | 77447 |
|  | 675 |  | 3 | 23560 | 38488 | 134788 | 220198 |
|  | 725 |  | 6 | 32552 | 49511 | 212908 | 323831 |
|  | 750 |  | 8 | 20457 | 30078 | 95547 | 140482 |
|  | 825 |  | 14 | 10325 | 13801 | 76883 | 102764 |
|  | 900 |  | 16 | 23196 | 28421 | 148071 | 181423 |
|  | 1000 |  | 6 | 21063 | 23226 | 122863 | 135483 |
| **Mean of PRO-001 values (pooled values across all cohorts)** | | | | | **26910** | **-** | **151413** |
| **Ratio (PRO-001/literature)** | | | | | **217** | **-** | **139** |

AUC, area under the concentration-time curve; Cmax, maximum observed concentration; Inf, infusion.

1. Normalised to a dose of 500 mg/m^2^ assuming linear PK for both C_max_ and AUC.
2. Mean values were provided in the publication and are reported here; only doses of 350 mg/m^2^ and higher are reported here.
3. Reported in pmol/10^6^ cells; converted to µM using a conversion factor of 2.63.
4. Converted to µmoles per mg of tissue protein using the molecular weight of 503.137, then converted to µM using the density of protein (1.22 g/cm^3^ [28]), and multiplied by 1000 mL per L.
5. Normalised to a dose of 500 mg/m^2^ assuming linear PK for both C_max_ and AUC and accounting for the difference in molecular weight between NUC-1031 (580.4) and gemcitabine (263.2).
6. Converted to µmol/hr per mg of tissue protein using the molecular weight of 503.137, then converted to µM/hr using the density of tissue protein (1.22 g/cm^3^ [28]), and multiplied by 1000 mL per L.

**Supplementary Figure 1.** Chemical structures of NUC-1031 and gemcitabine


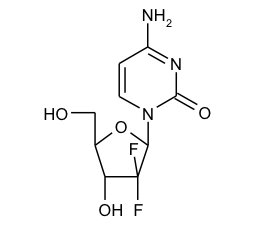


**NUC-1031**

**Gemcitabine**
